# Identification of key neuronal mechanisms triggered by dimethyl fumarate in SH-SY5Y human neuroblastoma cells through a metabolomic approach

**DOI:** 10.1007/s00204-024-03683-9

**Published:** 2024-02-18

**Authors:** Ana Margarida Araújo, Sandra I. Marques, Paula Guedes de Pinho, Helena Carmo, Félix Carvalho, João Pedro Silva

**Affiliations:** 1 Associate Laboratory i4HB, Institute for Health and Bioeconomy, Faculty of Pharmacy, University of Porto, 4050-313 Porto, Portugal; 2 UCIBIO, Laboratory of Toxicology, Department of Biological Sciences, Faculty of Pharmacy, University of Porto, 4050-313 Porto, Portugal; 3 Present Address: LAQV-REQUIMTE, Laboratory of Bromatology and Hydrology, Department of Chemical Sciences, Faculty of Pharmacy, University of Porto, 4050-313 Porto, Portugal

**Keywords:** Endometabolome, Neuroprotection, Nrf2 signaling, Neuronal metabolic pathways

## Abstract

Dimethyl fumarate (DMF) is an old drug used for psoriasis treatment that has recently been repurposed to treat relapse–remitting multiple sclerosis, mostly due to its neuro- and immunomodulatory actions. However, mining of a pharmacovigilance database recently ranked DMF as the second pharmaceutical most associated with cognitive adverse events. To our best knowledge, the signaling mechanisms underlying its therapeutic and neurotoxic outcomes remain mostly undisclosed. This work thus represents the first-hand assessment of DMF-induced metabolic changes in undifferentiated SH-SY5Y human neuroblastoma cells, through an untargeted metabolomic approach using gas chromatography–mass spectrometry (GC–MS). The endometabolome was analyzed following 24 h and 96 h of exposure to two pharmacologically relevant DMF concentrations (0.1 and 10 μM). None of these conditions significantly reduced metabolic activity (MTT reduction assay). Our data showed that 24 h-exposure to DMF at both concentrations tested mainly affected metabolic pathways involved in mitochondrial activity (e.g., citric acid cycle, de novo triacylglycerol biosynthesis), and the synthesis of catecholamines and serotonin by changing the levels of their respective precursors, namely phenylalanine (0.68-fold decrease for 10 μM DMF vs vehicle), and tryptophan (1.36-fold increase for 0.1 μM DMF vs vehicle). Interestingly, taurine, whose levels can be modulated via Nrf2 signaling (DMF’s primary target), emerged as a key mediator of DMF’s neuronal action, displaying a 3.86-fold increase and 0.27-fold decrease for 10 μM DMF at 24 h and 96 h, respectively. A 96 h-exposure to DMF seemed to mainly trigger pathways associated with glucose production (e.g., gluconeogenesis, glucose-alanine cycle, malate-aspartate shuttle), possibly related to the metabolism of DMF into monomethyl fumarate and its further conversion into glucose via activation of the citric acid cycle. Overall, our data contribute to improving the understanding of the events associated with neuronal exposure to DMF.

## Introduction

Dimethyl fumarate (DMF), the active principle of Tecfidera^®^, is an oral medication approved by the Food and Drug Administration (FDA) to reduce the activity and progression of relapse–remitting forms of multiple sclerosis (Blair 2019). In Germany, DMF is also present in a mixture of fumaric acid esters (Fumaderm^®^) widely used for psoriasis treatment (Venci and Gandhi 2013), and its safety profile has been assessed over 2 decades of pharmacovigilance (Mrowietz et al. 2009). DMF’s therapeutic action has been suggested to result from its degradation to the active metabolite monomethyl fumarate (MMF), which in turn up-regulates the Nuclear factor (erythroid-derived 2)-like 2 (Nrf2) pathway, often associated with a response to oxidative stress (Albrecht et al. 2012).

Recently, after mining the FDA Adverse Event Reporting System (FAERS) database, DMF was ranked as the second pharmaceutical most associated with cognitive adverse events (Andronis et al. 2020). A few cases of progressive multifocal leukoencephalopathy (PML), an opportunistic infection commonly associated with confusion, as well as impaired thinking, memory, and orientation, have also been reported in patients with multiple sclerosis being treated with DMF after they developed lymphopenia during the first year of DMF therapy (Lyons et al. 2022). However, DMF has also been associated with neuroprotective effects against cognition-related adverse events. For example, DMF has been shown to ameliorate cognitive impairment (indicated by a reduction in escape latency and path in the Morris water maze test) in mice with acute lung injury (Wang et al. 2021). Moreover, DMF treatment improved memory and learning (Morris water maze test) in hypothyroid rats (Pan et al. 2022), and reduced sepsis-associated inflammation, oxidative stress, and cognitive impairment in the brain tissue of a cecal ligation and puncture rat model (Zarbato et al. 2018). Altogether, these conflicting data concerning the association of DMF with cognitive adverse events urge the clarification of both the putative therapeutic and neurotoxic pathways activated by pharmacologically relevant doses of DMF, which, to the best of our knowledge, remain mainly undisclosed. As such, we herein aimed at ascertaining the metabolic alterations promoted by pharmacologically relevant concentrations of DMF in SH-SY5Y human neuroblastoma cells, through a non-target metabolomic approach using gas chromatography–mass spectrometry (GC–MS).

## Materials and methods

### Chemicals and materials

All the reagents used were analytical grade or of the highest grade available. Dimethyl sulfoxide (DMSO), Dulbecco’s modified Eagle’s medium (DMEM) high glucose, methoxyamine hydrochloride (≥ 98%), *N*,*O*-bis(trimethylsilyl)trifluoroacetamide with 1% trimethylchlorosilane (BSTFA + 1% TMCS), sodium bicarbonate, sodium chloride (NaCl, ≥ 99.5%), trypan blue solution 0.4% (w/v), trypsin–EDTA solution, 3-(4,5-dimethyl-2-thiazolyl)-2,5-diphenyl-2H-tetrazolium bromide (MTT), dimethyl fumarate (DMF) and all standards used throughout the work were obtained from Sigma-Aldrich (St. Louis, Missouri, USA). An antibiotic solution of 10,000 U mL^−1^ penicillin/10,000 µg mL^−1^ streptomycin, Hanks’ balanced salt solution (HBSS), and heat-inactivated fetal bovine serum (FBS) were obtained from Gibco Invitrogen (Barcelona, Spain). Methanol (≥ 99.9%), chloroform (≥ 99.8%), and pyridine (≥ 99%) were obtained from VWR (Leuven, Belgium). All sterile plastic material was obtained from Corning Costar (United States of America).

### Cell culture and drug exposure

Undifferentiated SH-SY5Y human neuroblastoma cells, obtained from the American Type Culture Collection (ATCC^®^ CRL-2266, Manassas, VA, USA), were grown in DMEM medium supplemented with 10% FBS and 1% of an antibiotic solution (100 U mL^−1^ penicillin and 100 μg mL^−1^ streptomycin). Cells were grown in 75 cm^2^ flasks and incubated at 37 °C under a humidified atmosphere with 5% CO_2_ until they reached 80–90% confluence. SH-SY5Y cells were trypsinized with a 0.25% trypsin/EDTA solution, counted following trypan blue staining, and the cell suspension was seeded in 96-well or 6-well plates (for cell viability and metabolomic assays, respectively) at the density of 50,000 cells/cm^2^. After seeding, cells were maintained at 37 °C, 5% CO_2_, for 24 h to allow proper surface reattachment. The medium was then discarded and replaced by fresh supplemented DMEM medium. Subsequently, cells were exposed to 0.1 or 10 µM DMF for 24 or 96 h. For the longer exposure (96 h), the cell culture medium was changed after two days and the cells were incubated with DMF for additional 48 h. A vehicle control, consisting of 0.1% DMSO, was also prepared. As a control, cells were seeded in a fresh medium without any treatment. All cell extracts used in the metabolomic studies were obtained from cells from five consecutive passages (23–27 passages). For cell viability evaluation, three independent assays (*n* = 3), with duplicates, were used, while for metabolomic studies, five independent assays (*n* = 5) were performed.

### Cell viability

The viability of SH-SY5Y cells was assessed by the MTT reduction assay, as previously described (Alexandre et al. 2020). This colorimetric assay is based on the mitochondrial reduction of the tetrazolium salt, with subsequent formazan formation. Briefly, after cells’ exposure to DMF or vehicle (0.1% DMSO), the cell culture medium was removed, and the cells were incubated for 2 h at 37 °C, 5% CO_2_, with fresh DMEM medium containing 500 μg mL^−1^ MTT. At the end of this period, the medium was removed and 200 µL of DMSO were added. The plate was shaken for 15 min in an orbital shaker (120 rpm) until the total dissolution of the formazan crystals, and the absorbance was measured at 550 nm in a multiwell plate reader (Biotech Synergy HT, Winooski, VT, USA). Results are expressed as the percentage of MTT reduction relative to the untreated cells at 24 h.

Statistical analysis of the MTT reduction assay was performed using GraphPad Prism 8 software (GraphPad Software, La Jolla, CA, USA). The normality of data distribution was assessed using the Anderson–Darling, D’Agostino–Pearson, and Shapiro–Wilk normality tests, and considering the acceptability of skewness and kurtosis values. Based on the normality results, a one-way ANOVA, followed by a Dunnett’s post hoc test was performed for each timepoint.

### Intracellular metabolic profile analysis

#### Sample extraction

Samples used for the analysis of the intracellular metabolome (or endometabolome) were collected and extracted according to a protocol previously optimized by our group (Araujo et al. 2018). Briefly, after cells’ exposure to DMF or vehicle, the medium was removed from the 6-well plates and the wells were washed twice with 0.9% NaCl to eliminate residual medium possibly containing extracellular metabolites. Then, 0.9 mL of an ice-cold methanol:water:chloroform solution (2:5:2, v/v/v) were added to each well to immediately quench cellular metabolism and extract the intracellular metabolites. Cells were scraped, transferred to a falcon tube, and sonicated on ice for 30 s to amplify the extraction efficiency. Then, samples were centrifuged (3000 g, 10 min, 4 °C) and the supernatant was collected into a glass vial. Quality control (QC) samples were prepared by pooling equal volumes of each sample used in the study and dividing them into multiple aliquots to avoid freeze–thaw cycles (Leon et al. 2013). All samples were kept at -80 ºC until analysis by GC–MS.

### Sample preparation

The extracts were thawed, vortexed, and carefully dried under a nitrogen stream. Then, a two-step derivatization process was performed, as reported by Araujo et al. (2018). Prior to GC–MS analysis, the dried extract was subjected to oximation by adding 50 µL of a methoxyamine solution (15 mg/mL methoxyamine hydrochloride in pyridine, 60 min at 70 °C) followed by a trimethylsilyl (TMS) derivatization using a mixture of BSTFA with 1% TCMS (room temperature, 60 min). After derivatization, 2 µL of TMS derivatives were directly injected into the GC–MS system. Sample preparation and injection were randomized to avoid analytical bias.

### GC–MS data acquisition and pre-processing

As also described by Araujo et al. (2018), the intracellular metabolic profile was evaluated in an EVOQ 436 GC system (Bruker Daltonics, Fremont, CA) coupled to an SCION Triple Quadrupole (TQ) mass detector using a Bruker MS workstation software (version 8.2, Bruker Daltonics, Bremen, Germany). Analyses were performed on a fused silica capillary column Rxi-5Sil MS (30 m × 0.25 mm × 0.25 µm; Restek Corporation, U.S., Bellefonte, Pennsylvania) using helium C-60 (Gasin, Portugal) as carrier gas (constant flow of 1.0 mL/min). The oven temperature was maintained at 70 °C for 2 min, rising to 250 °C at 15 °C/min (holding for 2 min), followed by an increase to 300 °C at a rate of 10 °C/min (held for 5 min). The injector temperature was 250 °C. The MS detector was operated in the electron impact (EI) mode at 70 eV and a split mode ratio of 1:20 was considered. The EI temperature was 270 °C, with a manifold temperature of 40 °C and 280 °C in the transfer line. Data acquisition was performed in full scan mode with a mass range between 50 and 500 m/z. A QC sample was repeatedly analyzed every nine samples under the same conditions.

For data pre-processing, all GC–MS chromatograms were converted into the CDF file format using the MASSTransit 3.0.1.16 software (Palisade Corp, Newfield, NY) and pre-processed using the MZmine 2.23 software (Pluskal et al. 2010). The pre-processing steps included crop filtering (m/z range 50–650 and RT range 6.00–24.50 min), baseline correction (asymmetric baseline corrector), peak detection (noise level 6.0 × 10^4^), deconvolution (minimum peak height 1.5 × 10^5^, baseline level 6.0 × 10^4^, peak duration range 0.02–0.21 min), and alignment (m/z tolerance 0.05, RT tolerance 0.05). After pre-processing steps, data were normalized by total chromatogram area and log-transformed to eliminate systematic bias. Artifact peaks from the chromatographic column, chromatographic peaks with a signal-to-noise of less than 3, as well as all peaks with relative standard deviation (RSD) higher than 50% across all QCs were manually removed from the data matrix.

### Multivariate and univariate statistical analyses

Multivariate analysis, namely principal component analysis (PCA) and partial least-squares discriminant analysis (PLS-DA), were performed using the SIMCA-P 13.0.3 software (Umetrics Umea, Sweden) on the data matrix scaled to unit variance. The robustness of PLS-DA models was assessed through the default method of sevenfold cross-validation, based on the R^2^ (goodness-of-fit parameter) and Q^2^ (predictive ability parameter) values, with values close to 1 indicating a robust model (Wheelock and Wheelock 2013). Notably, it is generally accepted that a Q^2^ value greater than 0.5 is considered satisfactory. Additionally, the permutation tests (500 permutations) were used to validate each PLS-DA model. As proposed by Wheelock and Wheelock (2013), the combination of p(corr) and variable importance to the projection (VIP value) of each m/z-RT pair present in the PLS-DA loading plot was used to extract the variables that contributed to the separation of the groups (|p(corr)|> 0.5 and VIP > 1). To provide a quantitative measurement of metabolic variations, normalized signal areas of the relevant metabolites (|p(corr)|> 0.5 and VIP > 1) obtained in each pairwise comparison were then used as input in the R Studio software (version 2021.09.0), to graphically represent the results and perform the univariate analysis. *p*-values were adjusted using the false discovery rate (FDR) correction, according to Benjamini and Hochberg (1995). Furthermore, for each potentially discriminant metabolite, the fold change was also determined.

### Identification process

The identification procedure was done as recommended by the Metabolomics Standard Initiative (MSI) (Sumner et al. 2007; Viant et al. 2017). A comparison of the mass spectrum of the unknown metabolite with the mass spectra present in the National Institute of Standards and Technology (NIST14) database was performed to identify the discriminant intracellular metabolites (|p(corr)|> 0.5 and VIP > 1). The retention index (RI) of each metabolite was also estimated according to the retention times (RTs) obtained for the alkane series (C_8_–C_40_) injected under the same chromatographic conditions. Only forward and reverse percentages of a match of 70% or above were taken into consideration for the preliminary identification. Whenever possible, presumed identification was confirmed by comparing the retention time and mass spectra of the metabolite with the commercially available standard compound analyzed using the same chromatographic column and temperature program. Unidentified metabolites are listed throughout the paper as ‘Unk_RT_’, where RT denotes the metabolite’s retention time. Table 1 lists all discriminant intracellular metabolites identified among all pairwise comparisons. Further details on the identified metabolites are listed in Supplementary Information (Tables SI-1 and SI-2).

**Table 1:** Discriminant metabolites identified in the pairwise models according to the corresponding PLS-DA loading plots (VIP > 1 and |p(corr)|> 0.5)

Metabolite			HMDB ID^a^	KEGG ID^b^	ID level^c^	Class
*Glycolic acid*			HMDB00115	C03547	L1	Organic acids and derivatives
*Fumaric acid*			HMDB00134	C00122	L1	
*Malic acid*			HMDB00744	C00711	L1	
*2-Oxoglutaric acid*			HMDB00208	C00026	L1	
*Citric acid*			HMDB00094	C00158	L1	
*Taurine*			HMDB00251	C00245	L1	
*Threonine*			HMDB00167	C00188	L1	Amino acids, peptides and analogues
*Phenylalanine*			HMDB00159	C00079	L1	
*Tryptophan*			HMDB00929	C00078	L1	Indoles and derivatives
*Glycerol-3-phosphate*			HMDB00126	C00093	L2	Glycerophospholipid
*2-Palmitoylglycerol*			HMDB11533	–	L2	Glycerolipids
*Unk*_*13.037*_			–	–	L3	Carbohydrates and carbohydrate conjugates
*Unk*_*7.421*_			–	–	L4	Unknown
*Unk*_*10.734*_			–	–	L4	
*Unk*_*12.088*_			–	–	L4	
*Unk*_*13.623*_			–	–	L4	

^a^HMDB: Human Metabolome Database;

^b^KEGG: Kyoto Encyclopedia of Genes and Genomes;

^c^L1 (Level 1)—Metabolites unequivocally identified by standards; L2 (Level 2)—Putatively annotated compounds (i.e., identification was based only on the mass spectrum similarity with commercial spectral libraries, retention indexes, and reverse percentage of the match); L3 (Level 3)—Putatively characterized compound classes (i.e., spectral and/or physicochemical properties are consistent with a particular class of compounds); L4 (Level 4)—Unknown compounds (unidentified or unclassified metabolites can still be distinguished based on mass spectrum data) (Sumner et al. 2007; Viant et al. 2017)

### Metabolic enrichment analysis

The free web-based platform MetaboAnalyst 5.0 (http://www.metaboanalyst.ca) was used to identify relevant biological pathways linked to the metabolic alterations induced by DMF (Chong et al. 2018). For the enrichment analysis, only discriminatory metabolites with Human Metabolome Database (HMDB) codes were taken into account and searched against the Small Molecule Pathway Database (SMPDB).

## Results

### Dimethyl fumarate did not affect SH-SY5Y cells’ metabolic activity

**Fig. 1:**
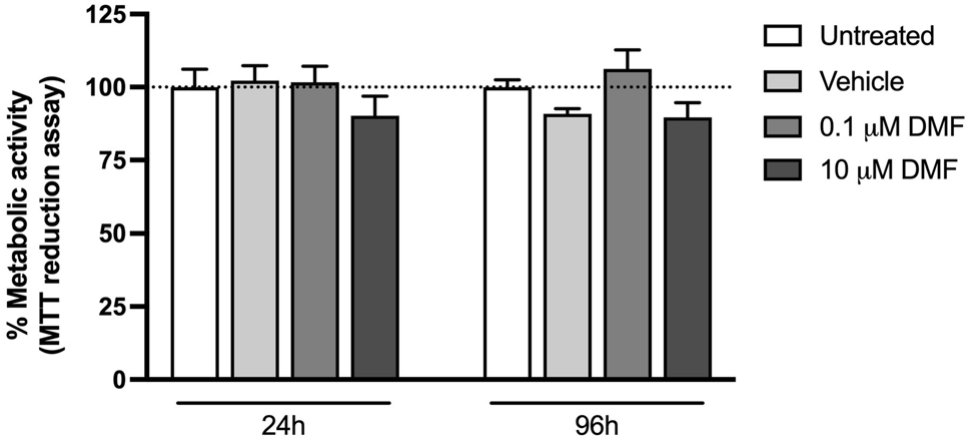
Metabolic activity, assessed by the MTT reduction assay, of SH-SY5Y cells exposed for 24 and 96 h to 0.1 μM and 10 μM dimethyl fumarate. Each bar represents the mean ± S.E.M., from at least three independent experiments, performed in duplicate. No statistically significant differences were found between the different concentrations tested and the respective vehicle control (0.1% DMSO), at each time point

DMF cytotoxicity was assessed using the MTT reduction assay. As observed in Fig. 1, an increase in the percentage of metabolic activity occurred for all conditions between 24 and 96 h, which was most likely due to regular cell proliferation. However, DMF did not alter the percentage of metabolic activity at any of the concentrations and time points tested, compared to the vehicle control, suggesting that this pharmaceutical, at these conditions, is not cytotoxic to the cells.

### Data quality assessment

GC–MS performance and method reproducibility were confirmed by unsupervised analysis (PCA) of data, including all samples under study and QCs. The data obtained showed a well-defined QC cluster, revealing that the chromatographic method has good reproducibility and that data are robust and suitable for further statistical analysis. The existence of possible metabolic differences between controls (only culture medium) and vehicle control (0.1% DMSO) was also evaluated. Multivariate results confirmed that there were no significant differences between these experimental conditions, since models (at 24 h and 96 h) showed extremely low accuracy and validity, as suggested by negative Q^2^ values (Q^2^ < − 0.1) and non-valid permutation tests (data not shown).

### Dimethyl fumarate modifies the SH-SY5Y cells’ intracellular metabolic profile

**Fig. 2:**
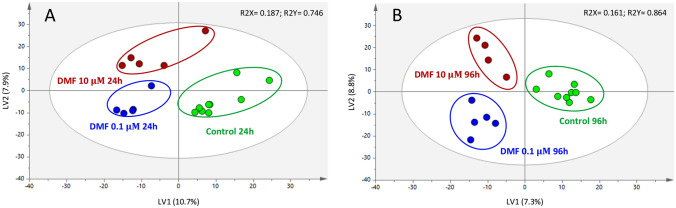
Partial least-squares discriminant analysis (PLS-DA) score scatter plots obtained for the DMF-exposed cells and controls (untreated and vehicle) after (**A**) 24 h and (**B**) 96 h. The ellipses indicate the 95% confidence limit of the model

PLS-DA models were built to improve the assessment of the intracellular metabolic differences between DMF and control groups (Fig. 2).

For both time periods, the models related to the highest concentration tested (10 μM) demonstrated a clear separation between exposed and control cells, having obtained a high predictive power (Q^2^ > 0.58) (Table 2). Furthermore, the permutation tests used to validate the PLS-DA models showed a good robustness level and predictive power of the models, without revealing overfitting, as the R^2^ and Q^2^ values of the permuted classes were lower than those of the original classes. Similar results were obtained for the lowest concentration (0.1 μM) after 24 h of exposure, with a high Q^2^ value (Q^2^ = 0.71). However, the 96-h model, while appearing to fit well to the data, exhibited a poor predictive ability (Q^2^ = 0.34), suggesting that the model suffered overfitting, i.e., it performed well on the observed data, but it was not able to generalize for new observations (Table 2).

**Table 2:** Cross-validation parameters obtained for each pairwise PLS-DA model

Time	PLS-DA model	R^2^X	R^2^Y	Q^2^	Permutation tests	
					R^2^	Q^2^
24 h	DMF 0.1 μM vs. Vehicle Control	0.353	0.990	0.710	0.990	0.433
24 h	DMF 10 μM vs. Vehicle Control	0.351	0.989	0.586	0.988	0.470
96 h	DMF 0.1 μM vs. Vehicle Control	0.283	0.992	0.339	0.993	0.423
96 h	DMF 10 μM vs. Vehicle Control	0.324	0.997	0.576	0.993	0.389

R^2^X—variance explained by the X matrix, i.e., GC–MS data; R^2^Y—variance explained by the Y matrix, i.e., sample class; Q^2^—predictive power; R^2^ and Q^2^ (permutation tests)—goodness of fit of the permuted model

**Fig. 3:**
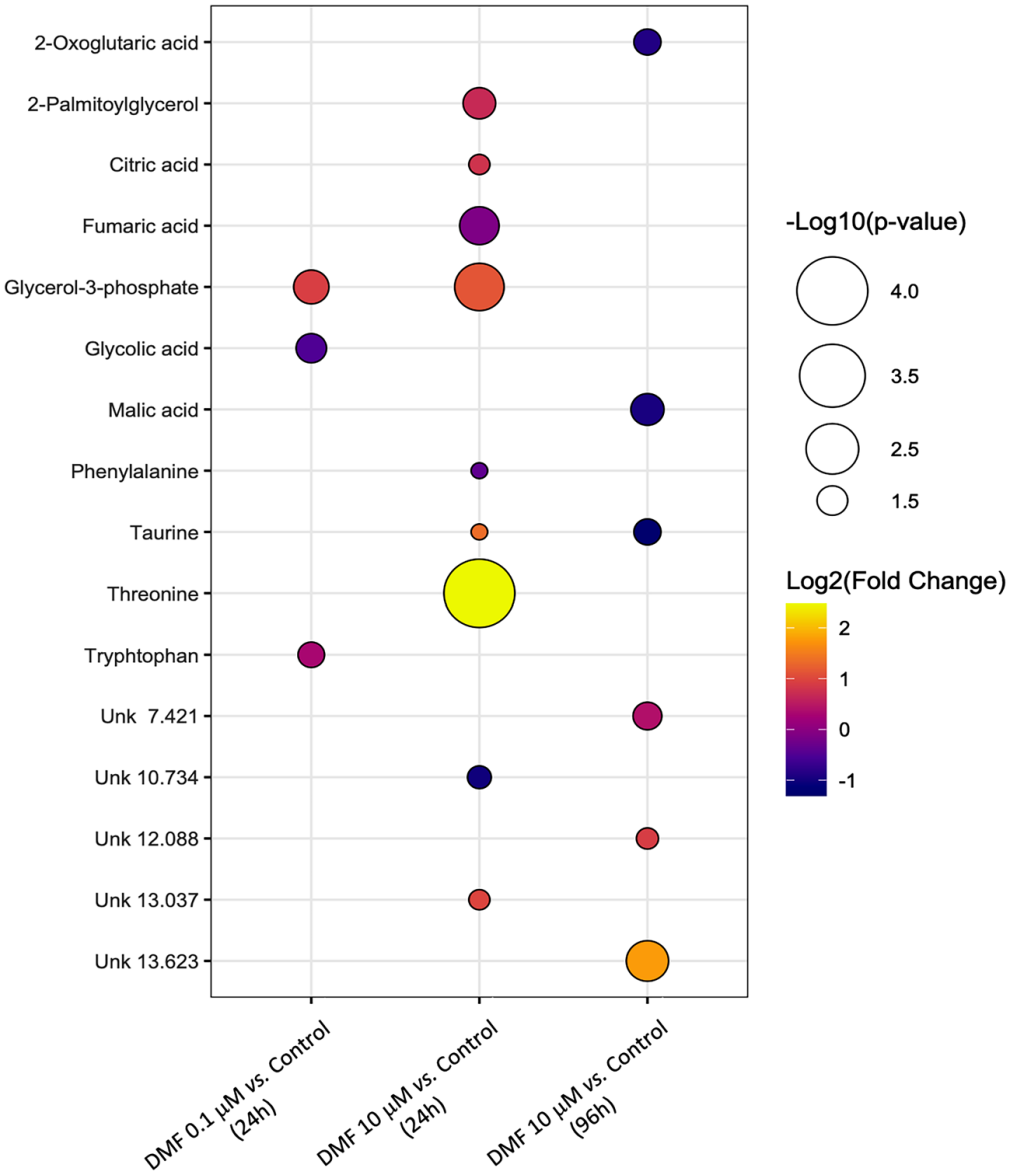
Bubble plot showing the magnitude and significance of alterations for each metabolite, according to the robust PLS-DA models obtained for dimethyl fumarate. Bubble size is proportionally related to the significance of the alteration, where smaller false discovery rate (fdr)-adjusted *p* values are associated with larger bubbles. The bubble color reflects the direction of the overall change (yellow for up-regulation and purple for downregulation)

Based on the analysis of loading plots for each pairwise analysis (0.1 μM DMF *vs.* control at 24 h and 10 μM DMF *vs.* control at 24 and 96 h), a total of 3, 9, and 6 discriminating metabolites, respectively, varied significantly as summarized in Fig. 3. The results showed that DMF affected the metabolic profile of neuronal cells exposed for 24 h even at the lowest concentration tested (0.1 μM), having significantly increased the levels of glycerol-3-phosphate and tryptophan and significantly decreased the level of glycolic acid. For the same time point, the overall impact caused by a 100-fold increase in concentration was considerably higher, with six metabolites appearing significantly increased (glycerol-3-phosphate, threonine, taurine, citric acid, 2-palmitoyl glycerol, and one unknown) and three metabolites significantly decreased (fumaric acid, phenylalanine, and one unknown). For the highest concentration tested (10 μM), an increase in the exposure time completely changed the neuronal metabolic profile when compared to acute exposure. In fact, taurine was the only common metabolite that changed after exposure to 10 μM DMF at both time points. Interestingly, the direction of the overall change was reversed, since a longer exposure to this drug led to a significant decrease of taurine in the SH-SY5Y cells. Alongside the decrease in taurine levels, there was also a significant decrease in two Krebs cycle intermediate metabolites (2-oxoglutaric acid and malic acid) and a significant increase in three unknown metabolites.

### Identification of altered metabolic pathways

**Fig. 4:**
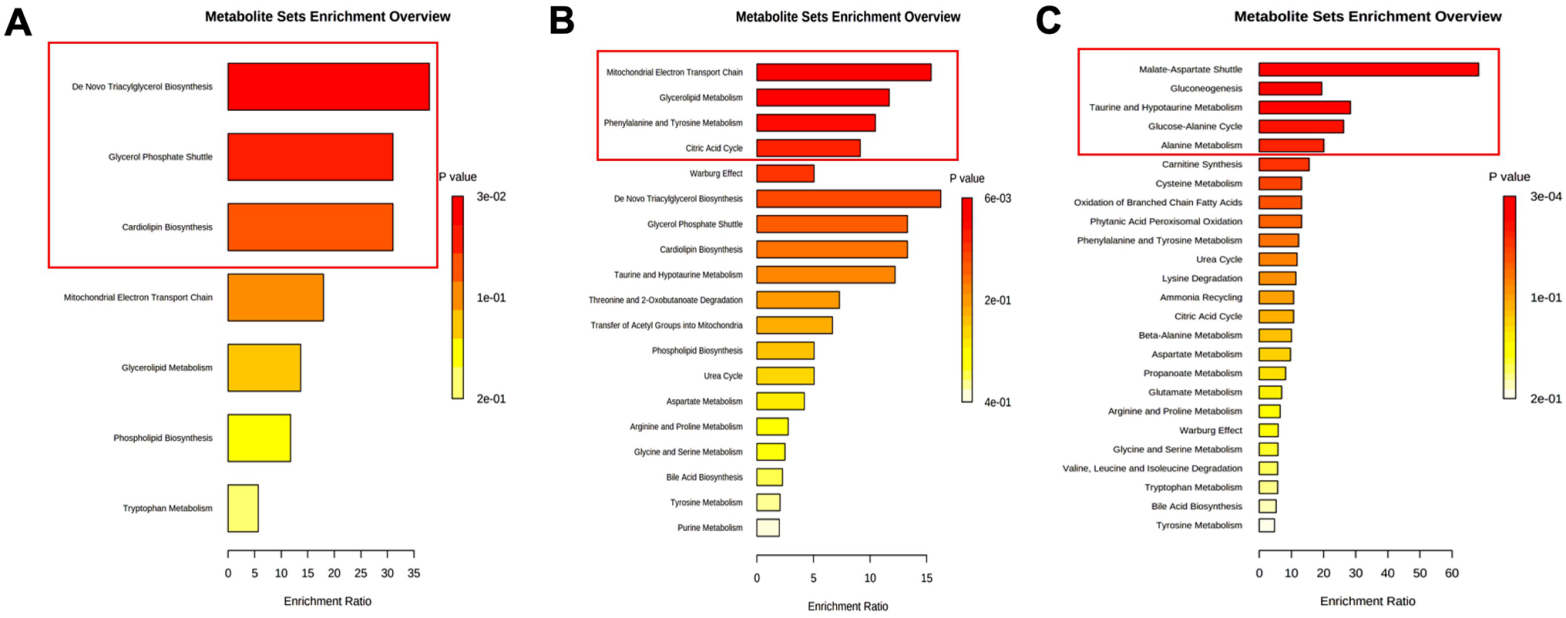
Overview of neuronal metabolic pathways altered by an acute exposure (24 h) with 0.1 μM (**A**) and 10 μM (**B**) DMF, and a prolonged exposure (96 h) with 10 μM (**C**) DMF. The colour of the scale is based on the *p* value, with yellow being associated with higher *p* values and red with lower *p* values. The red boxes highlight the significantly affected metabolic pathways (*p* < 0.05)

For an overview of the effects of exposure to DMF on SH-SY5Y cells and to identify key altered metabolic pathways, the discriminatory metabolites identified for this drug were analyzed using the MetaboAnalyst 5.0 software (Fig. 4A–C), considering the three pairwise combinations. Three of these combinations - 0.1 μM DMF vs. control at 24 h, and 10 μM DMF vs. control at 24 and 96 h - showed a high predictive power (Q^2^ > 0.58).

Our results demonstrated that for the lowest concentration/shorter incubation condition (0.1 μM at 24 h, Fig. 4A), the de novo triacylglycerol biosynthesis, the glycerol phosphate shuttle, and cardiolipin biosynthesis seemed to be significantly involved in the neuronal effects caused by DMF.

Acute exposure to DMF at the highest concentration tested (10 μM at 24 h, Fig. 4B) also significantly affected signaling pathways associated with the synthesis of triacylglycerols, like the glycerolipid metabolism, and other mitochondrial-related pathways, including the mitochondrial electron transport chain, and the citric acid cycle. Moreover, the metabolism of phenylalanine and tyrosine was also altered upon acute exposure to 10 μM DMF.

Considering the exposure for the longest period at the highest DMF concentration tested (10 μM at 96 h), DMF seemed to affect several biochemical pathways (compared to the previously described conditions), namely malate–aspartate shuttle, gluconeogenesis, taurine and hypotaurine metabolism, glucose–alanine cycle, and alanine metabolism (Fig. 4C).

## Discussion

DMF has been reported to have neuro- and immunomodulatory actions. However, the signaling mechanisms underlying its therapeutic and neurotoxic outcomes have not been fully disclosed so far.

Here, we first-hand showed that DMF altered the intracellular metabolic profile of neuronal cells at pharmacologically relevant concentrations. Considering that DMF did not change cell viability, as assessed by the MTT reduction assay, it is safe to assume that the observed metabolic changes did not result from its cytotoxic effects.

Our data indicate that the neuronal effects of DMF are concentration- and time-dependent, as changes in the intracellular metabolic profile of SH-SY5Y cells varied according to the concentration and time of exposure. Nevertheless, it should be taken into account that for the extended exposure (96 h), the cell culture medium was replaced and DMF added again at 48 h, which could have had an impact on the data observed at that time point.

Most of the metabolic pathways triggered by acute exposure (24 h) to DMF were associated with mitochondria (e.g., de novo triacylglycerol biosynthesis, electron transport chain, citric acid cycle), regardless of the concentration tested. For example, glycerol-3-phosphate levels were higher following DMF exposure than the vehicle control. The biosynthesis of glycerol-3-phosphate is a key regulator of mechanisms implicated in learning and memory. Moreover, defects in enzymes linked with the glycerol phosphate shuttle have been associated with neurological disorders and intellectual disability (Martano et al. 2016). Notably, glycerol-3-phosphate is required for the biosynthesis of triacylglycerols, namely cardiolipin and 2-palmitoylglycerol, two metabolites whose levels were also found elevated. Cardiolipin is a phospholipid uniquely present in mitochondria that plays a key role in the organization of mitochondrial membranes (Schlame and Greenberg 2017). Considering that reduced cardiolipin levels have been reportedly associated with cognitive deficits (Cole et al. 2018), DMF-mediated cardiolipin biosynthesis could possibly represent a neuroprotective mechanism. DMF also increased the levels of 2-palmitoylglycerol, which has been correlated with the increased activity of 2-arachidonoylglycerol, an endocannabinoid involved in a wide array of (patho)physiological functions, such as emotion, cognition, energy balance, pain sensation, and neuroinflammation (Baggelaar et al. 2018). In line with the activation of the de novo triacylglycerols biosynthesis, glycerolipid metabolism, which is required for the synthesis of triacylglycerols (Coleman and Mashek 2011), also appeared to be affected upon DMF exposure, as particularly noted by decreased glycolic acid levels.

Interestingly, glycerol-3-phosphate levels were altered after 24 h but not after 96 h of incubation with 10 µM DMF. Since this metabolite is associated with triacylglycerol biosynthesis, our data suggest that such synthesis of triacylglycerols is an early response to DMF, providing increased energy storage. As this study was performed in undifferentiated SH-SY5Y cells, which are metabolically active and may continue to divide, it is reasonable to expect that such stored energy is consumed over time (e.g., during cell division). In the exposure setting mimicking a semi-chronic DMF administration (96-h exposure), SH-SY5Y cells were incubated with DMF at the beginning of the assay and DMF was only added again at 48 h, meaning that cells were collected 48 h following the last addition of DMF. Based on our data, it is not possible to ascertain whether this second addition also resulted in increased glycerol-3-phosphate levels within the following 24 h (i.e., at 72 h). Nevertheless, since no changes were noted in glycerol-3-phosphate levels at 96 h, it is reasonable to assume that triacylglycerol biosynthesis is an early response to DMF.

Following the rapid conversion of DMF into MMF by the cells’ esterases, MMF has been described to enter into the citric acid cycle, being further metabolized in glucose, citric, and fumaric acid (CMPHU, 2013). As such, it is possible that the observed changes in the citric acid cycle in the presence of DMF may be actually attributed to its metabolite. However, rather than the observed decrease in fumaric acid levels, an increase would be expected. Of note, fumaric acid may also result from the metabolism of tyrosine, being often associated with decreased levels of catecholamines’ precursors, such as tyrosine and phenylalanine (CMPHU, 2013). Interestingly, acute exposure to 10 μM DMF decreased phenylalanine and increased tryptophan levels (compared to the vehicle), suggesting that it may simultaneously promote a decrease in the potential for catecholamine (e.g., dopamine, adrenaline) production and enhance serotonin formation, since phenylalanine is a precursor of catecholamines, and tryptophan is a precursor of serotonin (Dalangin et al. 2020). Importantly, it has been previously shown that changes in the levels of catecholamine and serotonin precursors influence their rates of conversion into neurotransmitters, possibly resulting in perturbations in brain function, including mood (e.g., depression, bipolar disorder) (Fernstrom and Fernstrom 2007; Walczak-Nowicka and Herbet 2021).

Of note, the genes that encode for most of the elements involved in these metabolic processes (e.g., citric acid cycle, triacylglycerol biosynthesis) may be directly regulated by the activation of Nrf2 (DeBlasi and DeNicola 2020), which is the primary target of DMF.

With the increase of taurine levels upon acute exposure (24 h) to DMF, but a decrease in these levels at a later time point (96 h), it is reasonable to expect that taurine could play a key regulatory role in the neuroprotective action of DMF. Taurine is a neuroprotective agent that plays several functions in the central nervous system, including the homeostasis of the electron transport chain, maintenance of glutathione stores, up-regulation of antioxidant responses, and modulation of intracellular calcium homeostasis (Baliou et al. 2021; Wu and Prentice 2010). DMF is an α,β-unsaturated carbonylic compound able to react with the sulfhydryl group of glutathione, a key modulator of the intracellular redox potential. In fact, this mechanism has been suggested to trigger the DMF-mediated inhibition of the translocation of nuclear factor kappa B (NF-κB) into the nucleus of human dermal fibroblast cells (Borgers et al. 2001). As such, it is possible that changes in glutathione levels could lead to an up-regulation of taurine levels to compensate for a loss in reduced glutathione. Supporting this hypothesis, intraperitoneal taurine administration to rats (50 mg/Kg) was reported to protect their heart tissue against nicotine-induced oxidative stress by promoting the normalization of glutathione stores (Şener et al. 2005). Moreover, Sun et al. (2018) recently observed that taurine protected SK-N-SH neuronal cells against the oxidative damage promoted by corticosterone via a mechanism involving the activation of the Nrf2 signaling pathway.

A longer exposure (96 h) to DMF seemed to mainly trigger pathways associated with glucose production, as suggested by the activation of gluconeogenesis and the glucose–alanine cycle. This glucose production-driven signaling was supported by the decrease in the levels of 2-oxoglutaric acid and malic acid, and the increased alanine metabolism. Indeed, citric acid cycle intermediates and some amino acids, like alanine, have been pointed to be glucogenic substrates by leading to the formation of oxaloacetate, which acts as a substrate for the glucogenic enzyme phosphoenolpyruvate carboxykinase, mediated by the citric acid cycle (Melkonian et al. 2023). The significant changes observed in the malate–aspartate shuttle further support this hypothesis. Indeed, this shuttle plays a crucial role in translocating oxaloacetate from the mitochondrial matrix (where it is produced), into the cytosol, for its conversion into phosphoenolpyruvate by the above-mentioned carboxykinase (Lu et al. 2008). Previously, Albrecht et al. (2012) observed that 10 μM DMF reached a maximum protective effect of primary cortical cultures and HT22 hippocampal cells from glutamate toxicity at 24 h, whereas MMF protective action developed slower and was only evident at 96 h. As such, considering the metabolism of DMF by intracellular esterases, it is likely that the metabolic changes observed at 96 h may actually result from MMF’s metabolism after it enters the citric acid cycle. However, Campolo et al. (2018) showed that MMF presented similar, but weaker, effects in vitro, compared to DMF, against α-amyloid-induced oxidative damage in both SH-SY5Y human neuroblastoma cells and organotypic hippocampal slice cultures, suggesting that the parent compound (DMF) may be the responsible for its in vitro effects. Still, it is worth noting that in vitro data may not reflect the effect of DMF in human patients’ cells, especially considering that MMF has been reported as the only active metabolite detected in serum and tissues following the oral intake of a Fumaraat120^®^ tablet (containing 120 mg of DMF and 95 mg of calcium-monoethyl fumarate) (Litjens et al. 2004).

## Conclusion

To our best knowledge, this work represents the first assessment of the intracellular metabolic profile of neuronal cells exposed to pharmacologically relevant concentrations of dimethyl fumarate, a drug commonly used for the treatment of psoriasis and relapse–remitting multiple sclerosis.

Our data showed a concentration- and time-dependent effect of DMF, with an acute cell response mainly linked to mitochondria-regulated pathways (e.g., citric acid cycle, de novo triacylglycerol biosynthesis), some of which (e.g., glycerol phosphate shuttle or cardiolipin biosynthesis) have already been associated with cognitive changes. Of note, most of such metabolic pathways have been shown to be directly modulated by Nrf2 activation, and have been clearly implicated in the cellular antioxidant response. Interestingly, we unravelled taurine as a potential key intermediate molecule in the antioxidant and neuroprotective action of DMF, as the levels of this compound, known for its essential role in maintaining redox homeostasis, have also been reported to be regulated by Nrf2 activation.

Moreover, DMF seemed to interfere with catecholamine (e.g., dopamine, adrenaline) and serotonin production by changing the levels of their respective precursors (e.g., phenylalanine, tryptophan), thus possibly affecting regular neurotransmission.

The possibility that MMF, rather than DMF, may be the main responsible for the observed effects, should also not be fully discarded. In vivo assays are thus required to further assess the extent of the neuromodulatory effects of DMF.

Nevertheless, our data represent an important contribution to a better understanding of the putative therapeutic and neurotoxic signaling pathways altered by DMF.

## Data Availability

Data generated or analysed during this study are included as Supplementary Material in this published article.
